# Exploring air pollution awareness: insights from interviews with technology enthusiasts using wearable monitoring devices

**DOI:** 10.3389/fpubh.2024.1468662

**Published:** 2024-11-27

**Authors:** Sara Bernasconi, Alessandra Angelucci, Stefano Canali, Andrea Aliverti

**Affiliations:** Dipartimento di Elettronica, Informazione e Bioingegneria, Politecnico di Milano, Milan, Italy

**Keywords:** air pollution awareness, wearable devices, personal air pollution exposure monitoring, interviews, technological interventions

## Abstract

This study aims to investigate shifts in awareness regarding air pollution and its correlation with interest in using wearable devices for air quality monitoring. 16 healthy participants, predominantly engineers, residing or studying in Milan, were interviewed to assess their knowledge and interest in air pollution. Participants then walked a predefined route of 4.5 km in Milan while observing real-time air pollution data recorded by a wrist-worn wearable device. Post-walk interviews explored changes in awareness and enthusiasm for personal air quality monitoring. Results indicated widespread recognition of pollution sources, including industries, transportation, and agricultural activities, and health effects. Interestingly, reliance on sensory cues for air quality evaluation was prevalent among participants, underscoring a potential bias toward olfactory indicators. Despite interest in personal air quality monitoring, concerns regarding continuous monitoring leading to feelings of powerlessness and mental stress were noted. Nevertheless, participants expressed interest in actionable information to mitigate these concerns and promote lifestyle choices to decrease exposure to pollutants. Notably, a shift in awareness was observed following interaction with the monitoring device, indicating its potential to enhance public awareness and support for air quality policies. Findings underscore the importance of technological interventions in promoting public awareness and understanding of air pollution dynamics.

## 1 Introduction

Promoting public awareness and a realistic perception of the risks associated with air pollution is pivotal for shaping individual behavior and promoting public support for air quality policies, ultimately contributing to public health improvement (1). Risk perception plays a crucial role in public response to environmental exposure. A perception of high risk, in addition to the perceived lack of control over the situation, leads to mental stress. On the other hand, a perception of low risk leads to no protective action and finally to higher exposure, with associated health hazards and no behavioral change to decrease the general level of pollution.

Considering this, public awareness must be elicited to improve public health and gain public support for policies aiming at making air quality healthier.

One of the main pillars that explains intentions and subsequent behavior is knowledge. In the realm of public knowledge about air pollution, and specifically about its sources, different considerations must be made. Firstly, the lay public seems to be more aware about the sources of pollution with respect to the specific pollutant, such as the prevalent use of the term “smoke,” as in the study on pupils in Greece (2). Pollution sources are also generally identified by the associated odor and are context-dependent and culture-dependent. For instance, in low-and middle-income countries the major source of indoor pollution comes from cooking. This pollution arises from the use of inefficient stoves and devices for cooking, which utilize fuels such as wood, coal, charcoal, dung, crop waste, and kerosene. Regrettably, millions of individuals continue to experience premature mortality annually due to household air pollution, as indicated by the WHO (3). In the end, definitions about air pollution and the elements identified as air pollutants lack universality, they differ between experts and laymen, but also between different populations in different contexts.

Even with a solid knowledge of pollution, individuals often perceive its presence primarily through sensory indicators like visibility and odor, or through health-related cues such as respiratory issues, particularly among those with asthma (4). Perception is additionally affected by the environmental quality and its physical characteristics; for instance, areas abundant in greenery are preferred, while areas like Birmingham with tall buildings tend to be associated with pollution (5). Moreover, cultural perceptions of landscapes play a role; as an example, London is commonly viewed as unclean, thereby influencing perceptions of the air quality as polluted (6). Perception is also influenced by comparative location-based evidence, especially for short-term travel, by comparative source-based evidence (e.g., opencast mining is less polluting with respect to other industries, such as steel), and by the context of place. In fact, it was found that if you are happy where you live, the general perception of pollution is influenced in a positive way. For instance, another example is the general belief that Beijing has cleaner air since all the efforts are made to improve the capital (7).

Another interesting effect in this regard is the ‘Halo effect’ (8). This effect is generally defined as a tendency for individuals to generalize their impressions of one attribute of an object to other attributes or the overall impression. In the context of air quality perception, this manifests as a widespread belief that one’s neighborhood is superior to others, despite lacking objective evidence to substantiate this claim (7).

Additionally, the experience of air pollution differs a lot, also in homogeneous groups (4). For instance, people with asthma experience specific health symptoms, given the same pollutants concentration of the air they are breathing. Different symptoms lead to different perceptions of air quality, which in turn creates a different attitude, the result of the evaluation of advantages and disadvantages of performing a certain behavior, according to the theory of planned behavior (9).

For what concerns the impact of air pollution on health, it was noted that this association is not obvious, maybe because respondents are uncomfortable to consider air quality responsible for poor health due to the lack of scientific knowledge (1). However, links are identified if pollution is more perceivable, and its physical impact is more acute. In addition, there is a rationalization of the fear of negative health impacts by considering other factors to contribute to poor health. In general, the lay public report non-specific health effects without entering details.

To cope with the concern about air pollution and its impact on health, different strategies take place such as denial, diminishment, and attribution, which are some standard strategies used for crisis response. Factors that alleviate public concern encompass perceptions of uncontrollability or powerlessness, the motivational crowding-out effects—indicating prioritization of other ‘major’ issues—, the perceived benefits associated with residing in polluted areas, such as employment opportunities, the perceived fairness denoting the belief in equal exposure among all individuals, the delayed manifestation of health effects, and acceptance. Furthermore, it is typically observed that individuals exhibit a phenomenon known as “optimistic bias,” which refers to the tendency for individuals to underestimate their own risk of experiencing negative events. Many people acknowledge the detrimental effects of environmental pollution but believe it will not personally affect them. This may inhibit pro-environmental action. According to a study investigating the impact of optimistic bias, it was found that it can be limited focusing on pro-environmental behavior with a wide range of impact (10).

Existing air quality communication strategies lack critical information including risk mitigation behaviors and long-term health impacts (11). Residents and expert stakeholders alike indicated a desire for specific information about acute health risks of daily air quality and long-term risks. Moreover, informants described feeling overwhelmed and powerless in the face of risk information without corresponding suggestions for strategies to mitigate health risks such as protective health behaviors. Introducing personal environmental monitors (12) that continuously sample air pollution concentrations, in addition to the already present solutions for air quality monitoring such as fixed stations, could help individuals become acquainted with pollution levels, potentially leading to a better understanding and acceptance of risk mitigation strategies. In fact, some studies in the indoor environment reported an effectiveness in awareness shifts, and subsequent behavioral change, when an intervention, i.e., showing indoor air quality values to participants (13, 14), was performed. An example is the project titled InformAria, which involved one of the authors, recently undertaken with the objective of creating tools to provide citizens with real-time air quality information, thus bolstering awareness, facilitating informed decision-making, and fostering sustainable urban development (15).

In the context of examining the effectiveness of real-time feedback on air pollution in promoting awareness shifts, to the best of the authors’ knowledge, only one previous study focused on a sample of technology enthusiasts (16). In this study, 22 participants used a portable device and its associated smartphone app for 1 month, receiving real-time feedback about air pollution. Surveys and interviews were conducted before and after the usage period. Participants were highly engaged with the topic and had financially invested in the technology’s development. Using technology enthusiasts as the initial test group can be advantageous, as they are less likely to encounter typical barriers to adopting new technology, enabling a more direct investigation of the device’s and app’s effectiveness without other confounding factors.

In the pilot study presented in this paper, we investigated these topics in a sample of 16 highly educated technology enthusiasts. The aim of this pilot study was to investigate their potential awareness shifts about air pollution related to the use of a personal environmental monitor with the possibility of monitoring real-time concentration of pollution and their willingness to modify behavior accordingly. The participants were enrolled in an experimental campaign in which they could visualize real-time pollution levels during a 1 h and 30 min’ walk in Milan. They were interviewed before and after the experience to gather information about the general knowledge, interest and concern about air pollution and the willingness in take pro-environmental action.

In Section 2 the methodology followed during the acquisition and the analysis performed on the collected interviews is described, and in Section 3 and 4 results are, respectively, presented and discussed. Then, in Section 5 conclusions highlighting the key findings and future implementation are reported.

## 2 Materials and methods

### 2.1 Experimental protocol and instrumentation

The experimental protocol was approved by the Politecnico di Milano Ethical Committee (approval no 39/2022, approved in September 2022) (17).

16 healthy participants (mean age 26.9 ± 5.7, 12 men) were involved in the study. In the cohort, all participants live or study in Milan, and have an age ranging between 23 and 46 years old. Regarding education, all participants have at least a bachelor’s degree, with 14 of them holding degrees in engineering, and mostly with a biomedical engineering background. The other 2 participants either study or work in a technical university, with an architectural and humanistic background. Based on these features of our sample we see participants as representing technology enthusiasts, meaning individuals who have a keen interest in and passion for technology, who actively seek out information on the latest technological advancements and innovations, who follow trends, participate in technology-related events, and may even contribute to the development or testing of new technologies.

**Figure 1:**
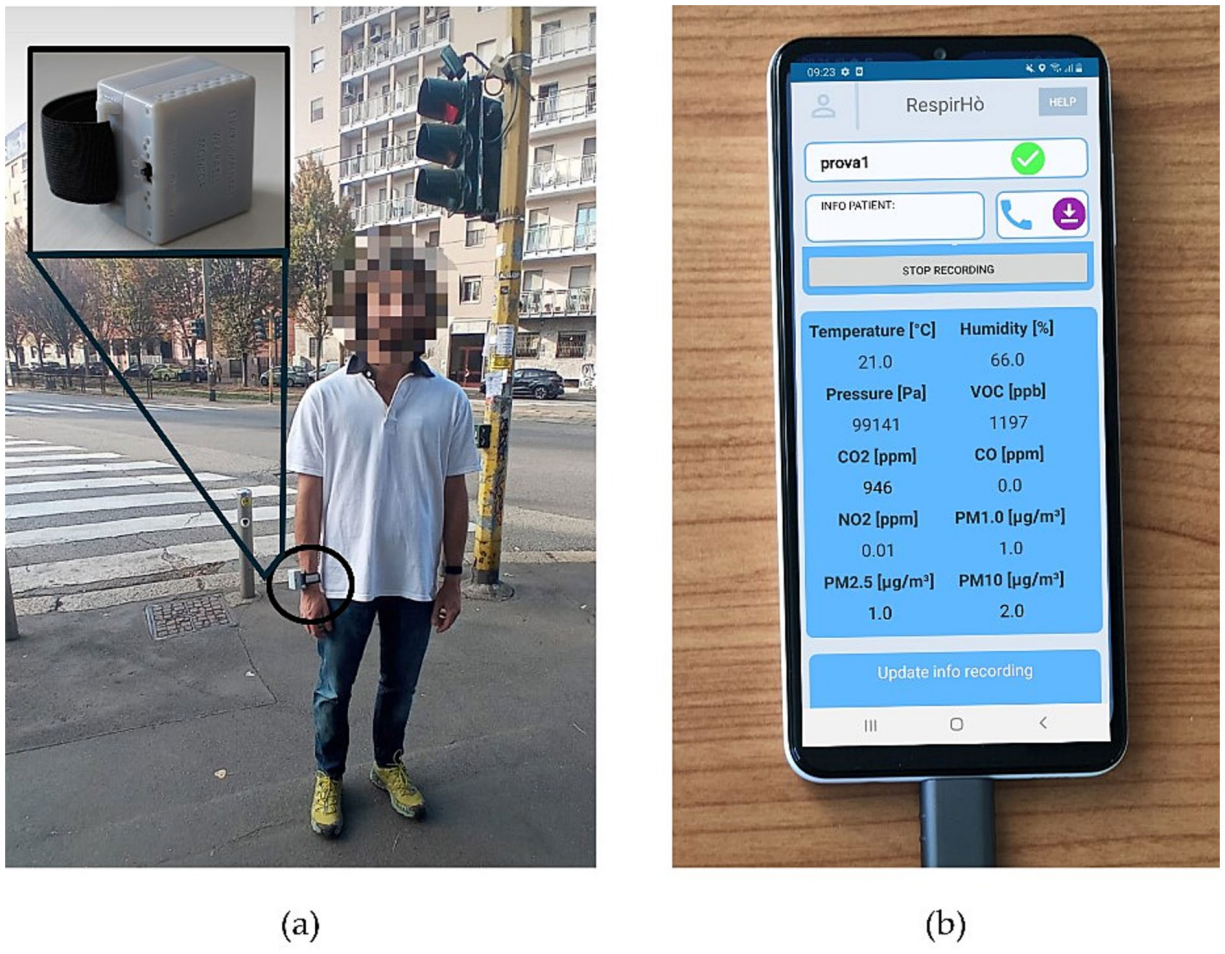
(a) Participant wearing the wrist-mounted environmental monitor, with a close-up view of the prototype; (b) Smartphone application displaying real-time pollution values to participants.

**Figure 2:**
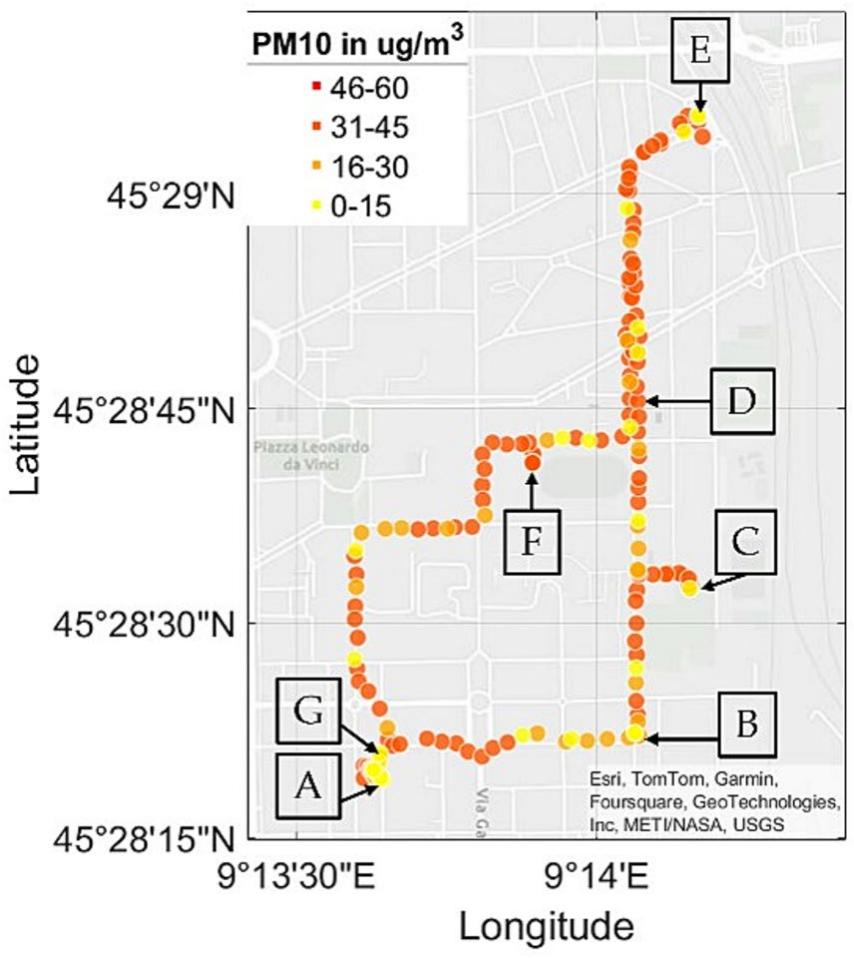
Acquisition path of a representative participant, displaying PM10 values. Letter ‘A’ corresponds to the indoor environment, letter ‘B’ to a gas station, letter ‘C’ to a park, letter ‘D’ to an intersection with traffic lights, letter ‘E’ to a train station, letter ‘F’ to a small park, and letter ‘G’ to a parking lot. Different concentrations of PM10 are represented with different colors.

The methodology comprises two phases of semi-structured interviews, designed to explore predefined topics while allowing participants the freedom to articulate additional insights based on their perceptions. The initial phase of interviews was conducted at the beginning of the trial, focusing primarily on investigating knowledge, perception, and interest. Then, participants were equipped with a newly designed wearable device that can monitor major pollutants and some indoor compounds that are harmful to health. The device, which is presented in another paper (18), is constituted by state-of-the-art commercial pollutant sensors and offers a high spatial and temporal resolution as a new packet of data is received each 24 s. It communicates through a wireless protocol with an application on the smartphone, which enables the subjects to watch pollution data in real-time, specifically particulate matter (PM1, PM2.5, PM10), nitrogen dioxide (NO_2_), carbon monoxide and carbon dioxide (CO, CO_2_), and total volatile organic compounds (TVOC). A picture of a participant wearing the device and the screenshots of what he could see in the app are shown in Figures 1a,b, respectively. The path, with values of PM10 of a representative participant, is reported in Figure 2. The walk started in an indoor environment (A), moving then near a gas station (B), a park (C), an intersection with traffic lights (D), a train station (E), a small park (F), in which a visible ARPA (*Agenzia Regionale per la Protezione dell’Ambiente*, Regional Environmental Protection Agency) fixed station is present, in a parking lot (G), and then returning to the initial indoor point. In the last phase of the protocol, participants were administered another semi-structured interview, with the aim of investigating possible shifts in awareness, in interest, perception and ultimately intentions of changing behavior according to the information that are now available.

### 2.2 Questions

In Table 1, the questions are presented, with their text translated from Italian.

**Table 1:** Interview questions.

Question ID	Question text (Translated from Italian)
Pre-1	Tell me the first 3 causes of pollution that come to your mind
Pre-2	Does air quality impact health? If yes, specify up to 3 possible health consequences of exposure to pollutants in your opinion.
Pre-3	What are the warning signs that make you think that the air in the surrounding environment is polluted?
Pre-4	Do you know if air quality monitoring is carried out and if it is possible to access that data? If yes, explain how the monitoring is done and where to find the data.
Pre-5	Would you be interested in having more information about the air quality around you?
Post-1	Do you think your awareness of pollution has changed after this walk? How?
Post-2	Do you think the warning signs you mentioned earlier are reliable for obtaining information about air quality?
Post-3	How interested would you be in having a device like the one used during the experimentation with you?
Post-4	Would it have an impact on your daily life to have continuous information about the air you are breathing? If yes, explain how; if no, explain why.
Post-5	If you could bring the device home, would you be interested in assessing indoor air quality? If yes, where would you measure it? (mention up to 3 points and why you are interested in those points) If no, why not?
Post-6	If you could take the device outdoors with you, would you be interested in evaluating outdoor air quality? If yes, where would you measure it? (mention up to 3 points and why you are interested in those points) If no, why not?

We began by delving into participants’ understanding of pollution sources, followed by exploring their awareness of the health effects associated with air pollution. If they acknowledged such impacts, they were encouraged to list up to three potential consequences of exposure. Additionally, we sought insights into their general perceptions of polluted air, limited to a maximum of three indicators. Their familiarity with air quality monitoring practices and whether they knew where to access relevant data were also investigated. Finally, participants were asked about their inclination toward seeking further information about air quality, allowing us to gauge their interest levels.

Following the walk, our initial inquiry aimed to capture any shifts in participants’ awareness. Subsequently, in the post-walk interview we evaluated their confidence in the pollution indices mentioned during the pre-walk interview. We then explored participants’ interest in utilizing a device like the one employed during data collection for personal air pollution monitoring. Additionally, we inquired whether they believed that continuous access to air quality information would influence their habits. Toward the conclusion, we asked participants if they desired to monitor air pollution indoors or outdoors, and if so, where they preferred to do so as another way to grasp interest in the subject.

All the interviews were recorded, transcribed, and then deleted after transcription.

### 2.3 Data analysis

A thematic analysis, based on Braun and Clarke’s qualitative research methodology (19), was conducted on the entire dataset. This systematic and inductive approach involves constructing hypotheses and categories directly from the data, a widely recognized method in the social sciences.

During the first phase interviews were transcribed and read by one of the authors. This phase enables familiarization with the dataset, during which notes were taken on interesting features and aspects that required specific consideration.

In the second and third phases, manual coding and theme association were performed by the same author. Manual coding of answers refers to the process of systematically categorizing and organizing the responses obtained from participants. In this method, interview responses are analyzed manually by researchers, who assign codes or labels to each response based on predetermined criteria or themes. These codes help to identify patterns, similarities, and differences in the data, allowing researchers to draw conclusions and insights from the interviews.

Categories able to collect different answers were identified and occurrences annotated. For instance, regarding pollution sources some answers were “cars,” “ships,” “traffic,” “transportation” and were all collected under the label “transportation.”

**Figure 3:**
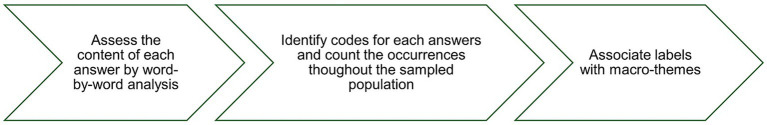
Scheme describing general steps of coding process.

Some macro-topics were highlighted, which are participants’ knowledge about air pollution, their interest, awareness shifts, and behavioral shifts. In the case of answers to the question about pollution sources, it was associated with the theme “participants knowledge about air pollution.” The coding process is illustrated in Figure 3.

The revision of the themes was then made by all the authors together, and other considerations emerged. All the authors had access to the transcribed text derived from the interviews.

In presenting the findings, a standardized approach was employed. For inquiries prompting participants to specify up to three options (Pre-1, Pre-2, Pre-3, Post-5, Post-6), data were depicted using a histogram, showcasing the frequency of various response categories. Additionally, a Venn diagram was employed to illustrate participants’ answers, with each response represented by a single point at the intersection of relevant categories. This approach enabled consideration of the combined responses from each participant.

In the case of open-ended questions (Pre-4, Post-1, Post-2, Post-3, Post-4), a pie chart was utilized, while responses to closed questions were detailed within the text. Some participant quotes are also reported to provide further insights into the topics under investigation.

An additional approach explored in this study was to examine whether there was a relationship between the levels of pollution to which participants were exposed and their responses to the post-walk questionnaire. The focus was specifically on PM2.5 exposure, as this pollutant exhibits significant spatial and temporal variability in outdoor environments, where participants spent most of the experimental protocol. Moreover, PM2.5 is known for its substantial health impacts and is well-recognized in the context of air quality studies. Median, maximum, and minimum PM2.5 exposure levels were calculated for the sampled population. Participants were then categorized into three equally sized groups based on median exposure: low exposure (≤15 μg/m^3^), medium exposure (15–30 μg/m^3^), and high exposure (>30 μg/m^3^). Post-walk responses were analyzed according to these exposure groups, with a focus on changes in awareness, interest in wearable air pollution monitoring devices, and willingness to modify habits based on pollution levels.

Additionally, we examined the responses in relation to the variability in exposure, defined as the difference between the maximum and minimum PM2.5 values. Participants were divided into three equally populated groups according to variability: low variability (0–35 μg/m^3^), medium variability (35–50 μg/m^3^), and high variability (50–110 μg/m^3^). The post-walk responses were then analyzed according to these variability groups.

## 3 Results

### 3.1 Pre-walk answers

**Figure 4:**
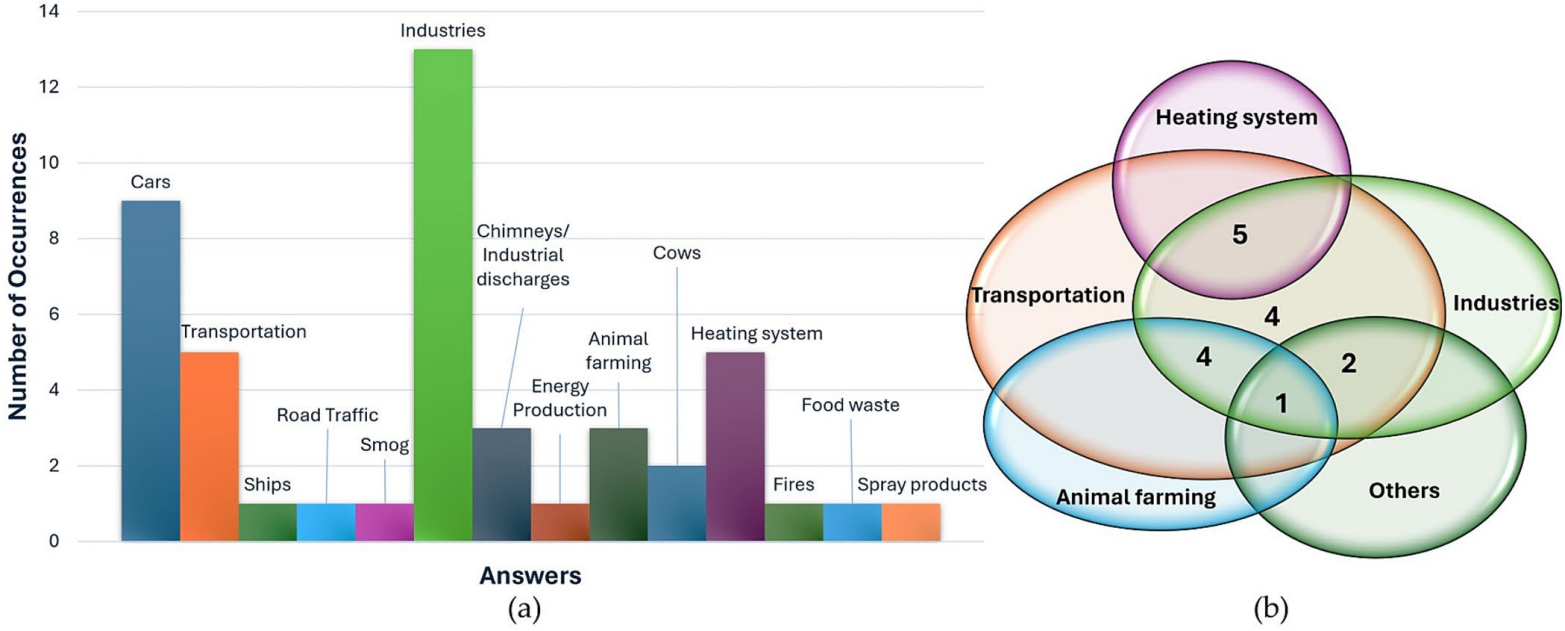
Answers to question Pre-1 “Tell me the first 3 causes of pollution that come to your mind,” represented through histogram of occurrences of responses (a) and Venn diagram (b).

Answers to the questions about the sources of pollution (Pre-1) reveal a generally good knowledge. 15 people out of 16 reported transportation, such as the use of cars and trains, as a source of pollution. 4 people cited intensive animal farming, 5 added heating systems. Wildfires, food waste, and the use of spray products were each reported only once by the entire population. In the diagram in Figure 4a, the occurrences of different answers are reported. Figure 4b shows a Venn diagram that considers the combinations of sources in each participant’s answers, with a maximum number of three answers. For example, it can be observed that 5 people reported as source of pollution the heating system, together with industries, and transportation, while 4 participants reported only transportation and industries. All the following Venn diagrams can be interpreted in the same fashion.

**Figure 5:**
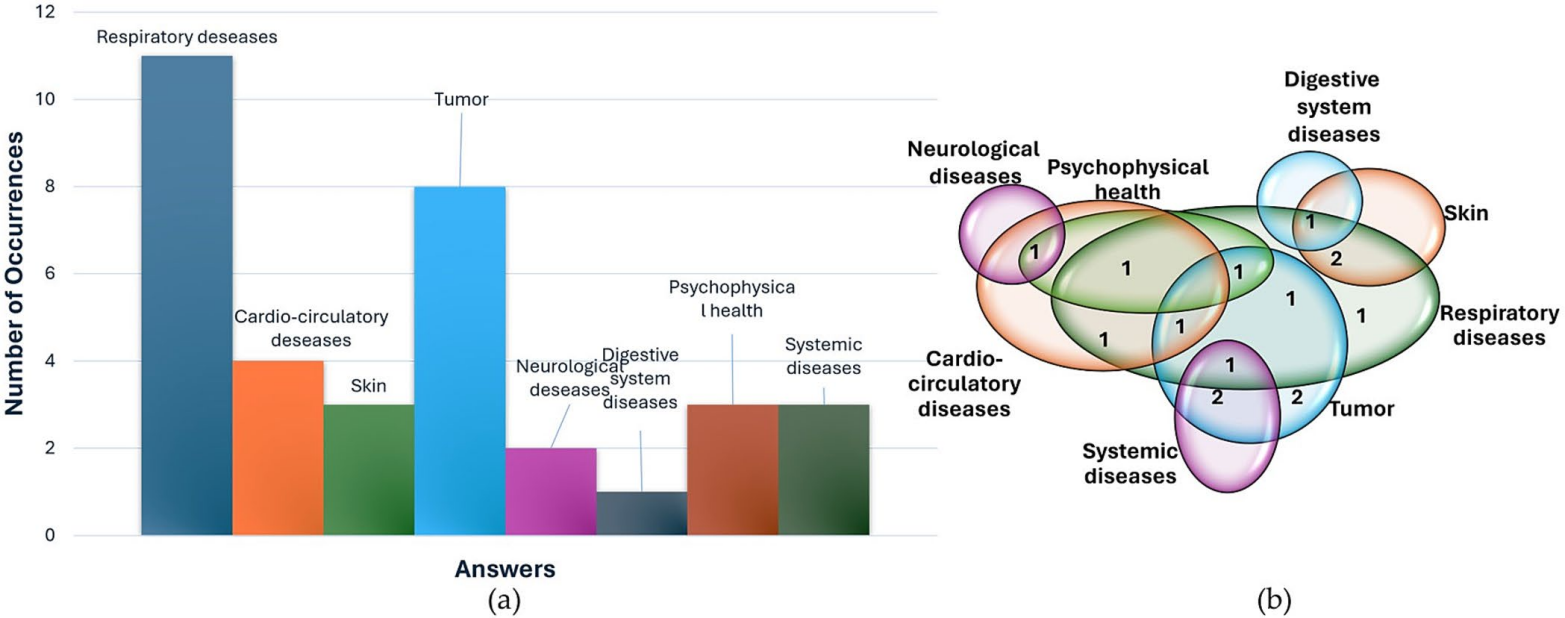
Answers to question Pre-2 “Does air quality impact health? If yes, specify up to 3 possible health consequences of exposure to pollutants in your opinion,” represented through histogram of occurrences of responses (a) and Venn diagram (b).

Regarding the health implications of air pollution (Pre-2), the most widely acknowledged effects pertain to respiratory issues and tumor development. 11 people out of 16 have referred to respiratory diseases, and 4 people cited cardio-circulatory diseases. 9 participants cited tumors, 3 skin diseases, psycho-physical health, and systemic diseases. Additionally, 2 of them cited neurological diseases, while 1 person cited allergies (included in the ‘respiratory diseases’ category) and problems to the digestive system. The histogram of responses and Venn diagram are reported in Figures 5a,b, respectively.

**Figure 6:**
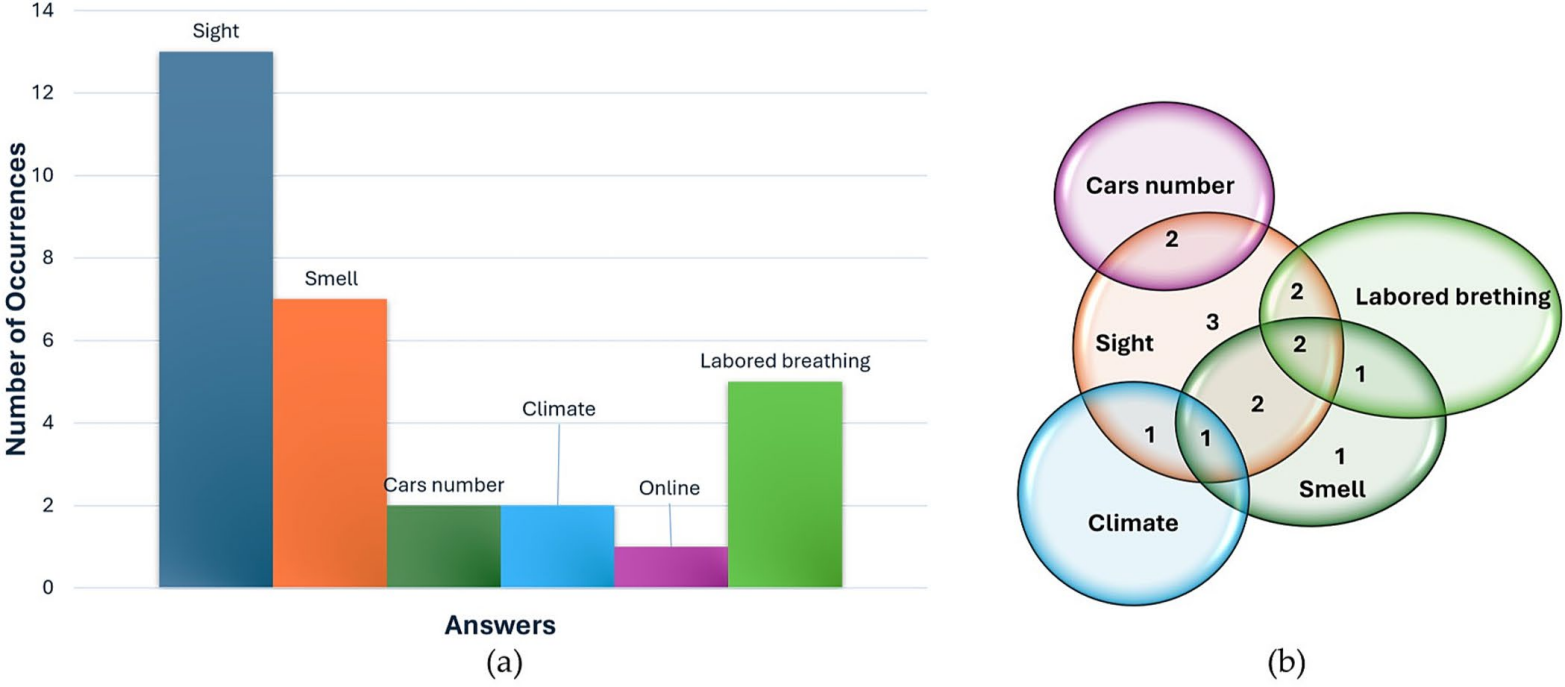
Answers to question Pre-3 “What are the warning signs that make you think that the air in the surrounding environment is polluted?” represented through histogram of occurrences of responses (a) and Venn diagram (b).

**Figure 7:**
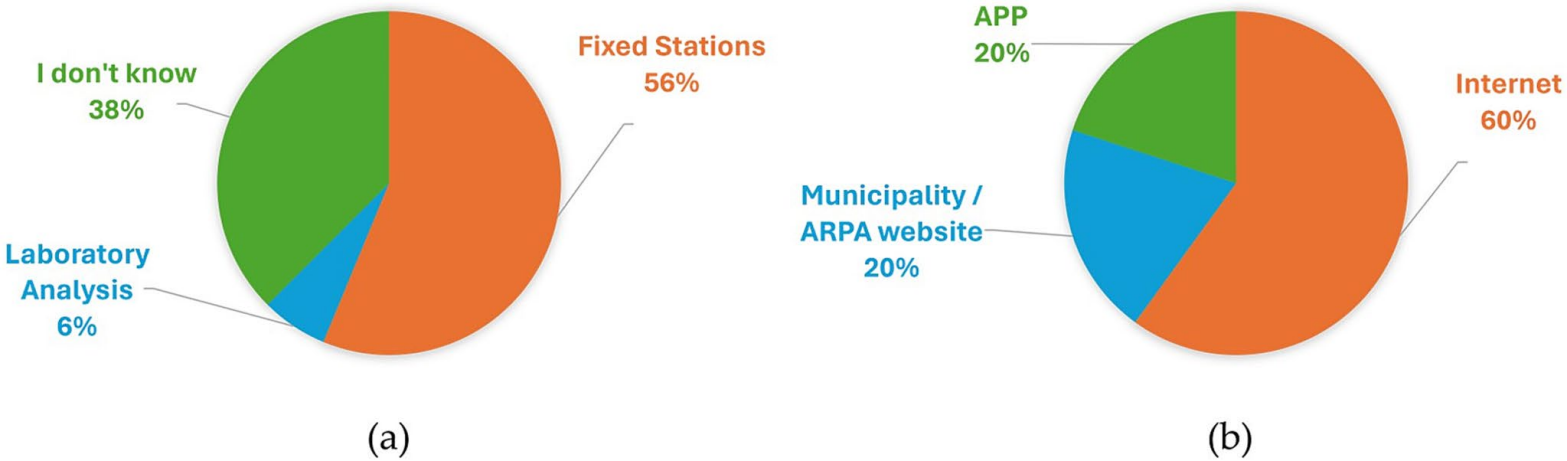
Answers to question Pre-4 “Do you know if air quality monitoring is carried out and if it’s possible to access that data? If yes, explain how the monitoring is done and where to find the data?” Monitoring technologies are reported in (a), while data sources are outlined in (b).

In terms of the indicators they consider informative for evaluating pollution (Pre-3), most respondents indicate visual assessment as their primary method for gaging air pollution. Specifically, 13 participants responded that they use vision to grasp information about air quality. Here, 2 sample answers are reported: “*…For example in Milan, […], you observe that there is a large amount of dust in the air and anyway often if there is dust there are other finer substances that you do not see.*” (participant 11, male, 25 years old), or “*The fact of seeing large deposits of dust on windows, balconies, filters, for example masks that after a day in Milan are black.”*(participant 18, male, 25 years old). 7 interviewees reported using smell- *“Coming to Milan, I immediately notice the shroud we have above us, based also on the weather and even the smell. On an olfactory level, therefore.*” (participant 6, female, 24 years old)- and 5 reported labored breathing as index of pollution. 2 people talked about the number of cars, while 2 talked about climate. Responses are collected in the histogram in Figure 6a and in the Venn diagram in Figure 6b. Overall, most participants indicated using sensory cues to assess air pollution and were also aware of the presence of monitoring technologies for air pollution (11 out of 16). The remaining respondents merely speculated, indicating a low level of interest, and understanding. 6 participants said they did not know anything about monitoring technologies, while 9 cited fixed stations as monitoring technology and 1 person mentioned laboratory analysis. Results are represented by the pie diagram in Figure 7a.

In terms of data availability (Pre-4), 5 individuals were unaware of where to access this information. Among the remaining 11 respondents, various sources were cited. Some provided generic responses, such as ‘on the internet,’ while three mentioned a ministerial website as a possible source. Additionally, others cited specific sources such as the ‘Weather’ app on iPhone (20), the ARPA website (21), and the popular website ‘ilMeteo’ (22). One participant reported “If you look at the ilMeteo app or the iPhone’s weather app, the air quality shows up, indicating the level it could be—whether it’s low, high, etc.” (participant 6, female, 24 years old). These results are represented by a pie diagram in Figure 7b.

We found substantial interest in obtaining further information about air quality (Pre-5), with 25% of respondents expressing absolute interest, 51% indicating interest, and an additional 19% expressing a desire for more information, albeit with certain conditions or concerns about potential drawbacks. The necessity to adapt to the situation, and the perceived powerlessness emerged. The idea of actionable information was explicitly mentioned by different participants. For example, participant 14 stated: “*Yes, especially if translated into practical terms. If I look at them and do nothing, that is one thing, but if instead I look at them and it tells me not to go out because it is polluted, that is fine.*” (participant 14, male, 27 years old). Moreover, some participants, like participant 7, expressed frustration about the lack of actionable information, and the only action he could think of to be less exposed was relocation “*Yes, I would be interested, but the thing is that for the current situation it would be information for its own sake because if I see that the air is polluted, it is not like I am going to move to the mountains.*” (participant 7, male, 32 years old).

### 3.2 Post-walk answers

**Figure 8:**
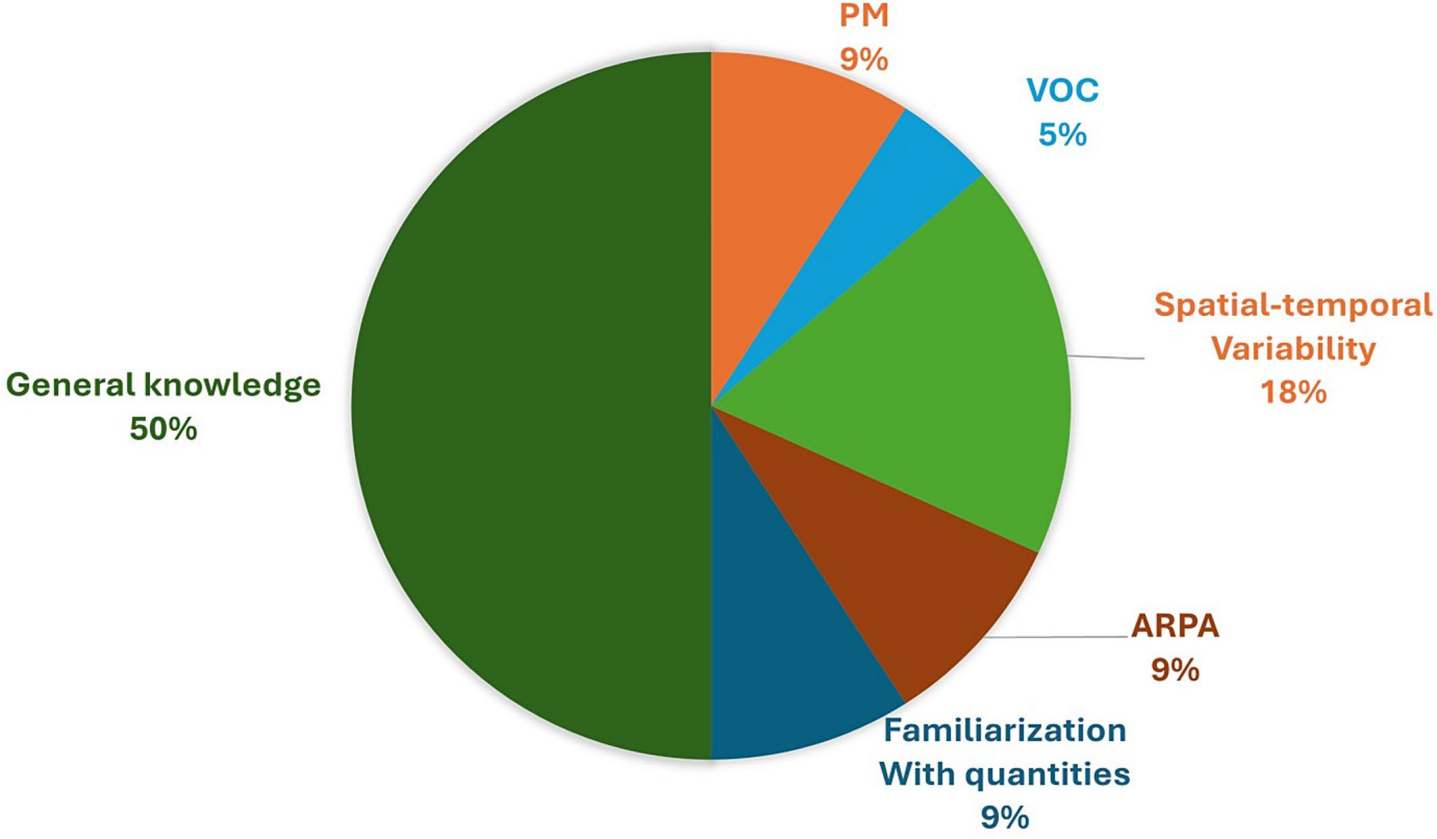
Pie diagram of answers to question Post-1 “Do you think your awareness of pollution has changed after this journey? How?”

Most of the participants reported an increased awareness (Post-1), after testing the personal air pollution monitor while walking for 4.5 km in Milan. 7 participants expressed absolute certainty, while an additional 7 reported moderate confidence in their increased awareness. Conversely, 2 participants indicated no change in their awareness level. As evidence supporting their claims, 4 individuals reported an increased awareness of the spatial–temporal variability of pollution: “*Yes, I had never considered movements, means of transportation, intersections… but now I have more in mind the values, the areas where it is more polluted, …*” (participant 16, male, 26 years old). 2 participants noted a greater familiarity with concentration values: “*Yes. I have quantified something that I previously sensed, suspected.*” (participant 11, male, 25 years old). The lack of actionable information was once again underlined: “*I have more knowledge, but I do not know how to use it.*” (participant 7, male, 32 years old). Answers are reported in the pie diagram in Figure 8.

About the reliability of the indices cited in the pre-walk interview (Post-2), 7 people affirmed to consider their perception of air quality as reliable with respect to the real concentrations, while 6 individuals viewed their perceptions as a starting point. Instead, 3 people considered perception as non-reliable, underlying the importance of quantifying: “*Of course not, they are very subjective and qualitative; instead, quantification is obviously necessary. Also, because perhaps one might associate a bad odor with air pollution, but there is no logical connection. So, obviously, measurement is necessary.*” (participant 12, female, 46 years old).

**Figure 9:**
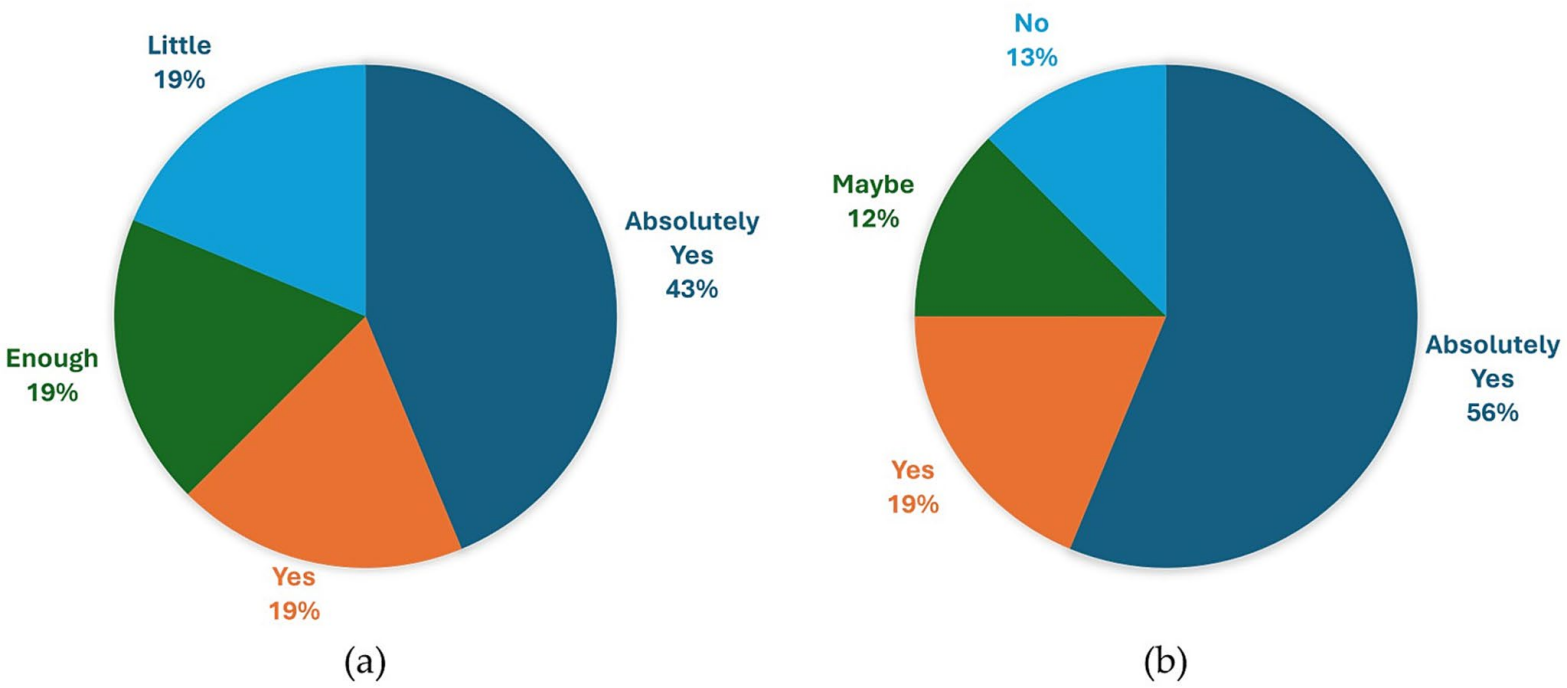
Pie diagrams of answers to question Post-3 “How interested would you be in having a device like the one used during the experimentation with you?” (a) and Post-4 “Would it have an impact on your daily life to have continuous information about the air you are breathing? If yes, explain how; if no, explain why” (b).

The evaluation of interest in owning a personal environmental monitor (Post-3), reported in Figure 9a, has revealed several noteworthy insights. 7 individuals express enthusiasm for the device, 3 show definite interest, 3 indicate moderate interest, while the remaining 3 exhibit minimal interest. Additionally, some individuals have raised concerns regarding over-monitoring: “*A lot, but I would become obsessed.*” (participant 7, male, 32 years old) and “*So and so. Then, I do not really like having constant info, but at least using it once in the area where you live should be a right of everyone*” (participant 11, male, 25 years old). Furthermore, the topic of actionable information or lack thereof emerged again: “… *for my general activities, it does not change my choices, I’m not so sensitive as to not go to a place because the air is not good.* “(participant 12, female, 46 years old) and “*If this translates into a change in my habits, then yes, I am very interested in having it on my wrist.*” (participant 14, male, 27 years old). A participant also highlighted the necessity for a substantial number of individuals to opt for wearing the device to gather sufficient data for meaningful assessment of pollution levels in an area.

In Figure 9b, a pie diagram of the answers to the question about the willingness to change behavior according to pollution (Post-4) is reported. 9 people reported that they would surely change their habits if they know the level of pollution, changing the place where they play sport outdoors, or changing the attended place for study, or ventilate the rooms or, at the extreme, change the place where to live. 3 participants indicated a willingness to modify their habits, 2 expressed uncertainties regarding potential changes, and 2 reported no intention to alter their behaviors. The concern about over-monitoring was reported: “*Yes, it would have an impact, but, as I have already explained, having too much information that you already know creates more stress than anything else. Having it initially to make decisions and, if necessary, relocate [is fine], but having it every day, in my opinion, is excessive.*” (participant 11, male, 25 years old). A participant also reported the importance of the reliability of data to change behavior accordingly.

**Figure 10:**
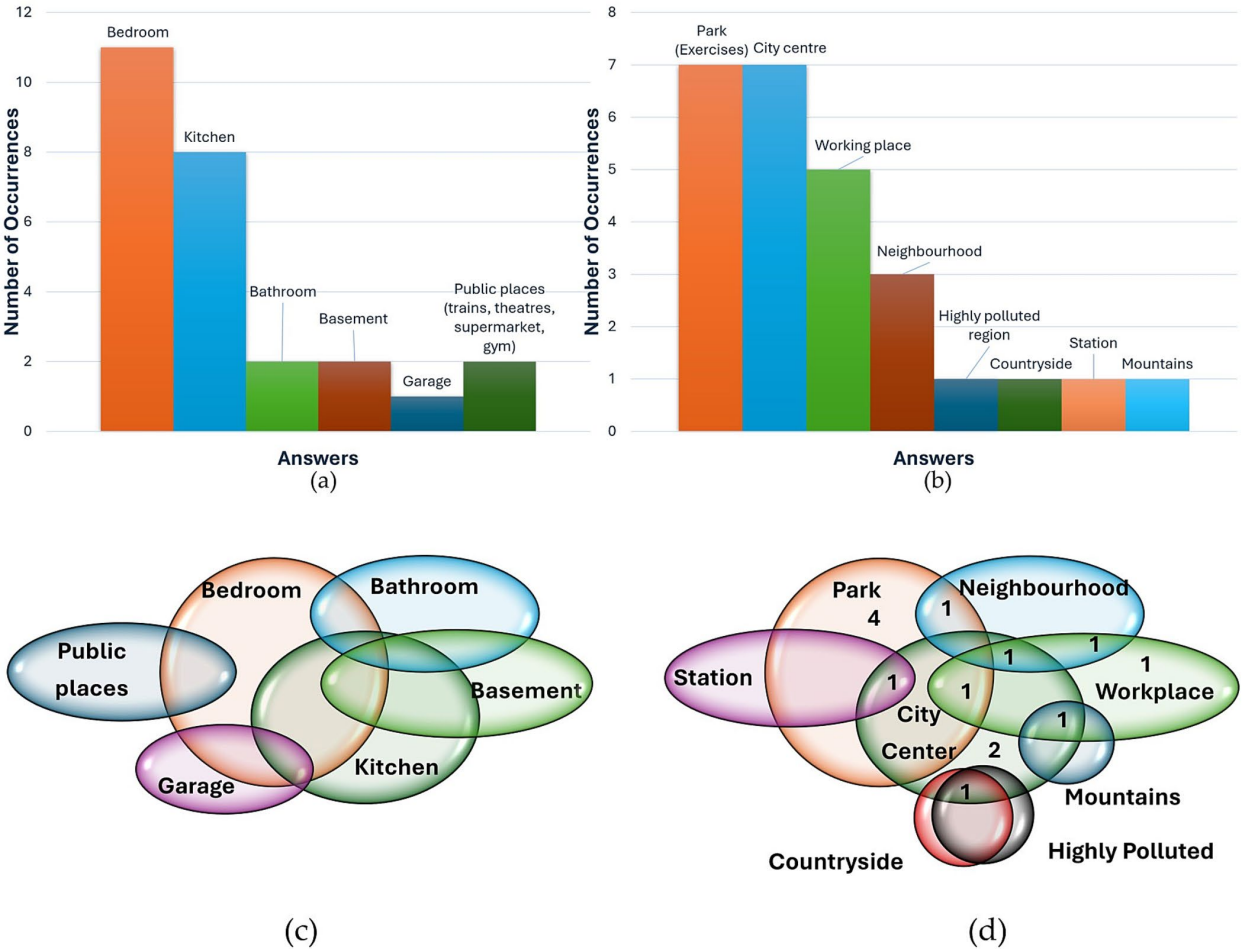
Answers to question Post-5 “If you could bring the device home, would you be interested in assessing indoor air quality? If yes, where would you measure it? (mention up to 3 points and why you are interested in those points) If no, why not?” and Post-6 “If you could take the device outdoors with you, would you be interested in evaluating outdoor air quality? If yes, where would you measure it? (mention up to 3 points and why you are interested in those points) If no, why not?” Histogram of occurrences of responses to the two questions are, respectively, reported in (a) and (b), while Venn diagrams in (c) and (d).

The focus on continuous monitoring of indoor air pollution (Post-5) was centered on several key areas: the kitchen to assess emissions generated during cooking, living spaces such as bedrooms or dining rooms where occupants spend significant time, particularly while studying, with consideration for strategic ventilation, and the bedroom during nighttime hours. Additionally, there is a reported willingness to conduct spot measurements out of sporadic curiosity, such as assessing the efficacy of bathroom fans or monitoring air quality in consistently enclosed environments like garages. There is also an interest in monitoring public indoor environments such as theaters, trains, or gyms. The histogram and Venn diagram of these answers are reported in Figures 10a,c.

For what concerns outdoor environment (Post-6), participants expressed a willingness to monitor the area where they live and the vicinity of their workplace. Regarding this finding, it should be noted that participants did not specify whether the term “working area” referred to the indoor environment of their workplace or the outdoor area surrounding it. One frequently reported potential application of the device is determining suitable locations for engaging in physical exercise: “*The issue is that it might interest me, but if I have to be conditioned by this thing… then it depends on what you do afterward. If I have to decide which park to go to, yes.”* (participant 7, male, 32 years old). Also, participants expressed a willingness to monitor air pollution values in the city center, in the countryside, in the mountains to familiarize themselves with the absolute values and refine their perception of air quality. One participant also pointed out in the response to question Post-6 the need for data collection, facilitating analysis by others to glean insights. Subsequently, as awareness increases, the device would be employed to implement actions aimed at enhancing overall well-being: “*I would wear it to collect data that will then be analyzed by others to gain insights. Mainly at the beginning for this purpose, then when this perhaps turns into an awareness, I would wear the device to take those actions that would make me live a better life.*” (participant 14, male, 27 years old). The histogram and Venn diagram of these answers are reported in Figures 10b,d.

To evaluate the relationship between exposure and post-walk responses, Table 2 presents the minimum, maximum, and median PM2.5 exposure levels recorded for each participant during the protocol, and their exposure group and variability group. The parallel analysis of exposure groups, as outlined in the Methods section, indicates that consistent responses were observed only among the high exposure group. Specifically, no participant in this group reported a lack of change in awareness, unwillingness to change, or low interest in obtaining a wearable device for air pollution monitoring. Beyond these findings, no other consistent patterns emerged when comparing behavior across different exposure levels, nor was any pattern observed in the variability groups, including those with high variability.

**Table 2:** PM2.5 concentrations, exposure, and variability group for each participant.

Participant ID	Min [μg/m^3^]	Max [μg/m^3^]	Median [μg/m^3^]	Exposure group	Variability group
P01	0.17	26.49	12.01	Low	Low
P02	2	40	22	Medium	Medium
P03	12	76	31	High	High
P04	5	40	19	Medium	Low
P05	10	49	22	Medium	Medium
P06	6	54	37	High	Medium
P07	8	68	44	High	High
P08	3	28	15	Low	Low
P09	1	110	25	Medium	High
P10	3	36	22	Medium	Low
P11	1	51	15	Low	Medium
P12	2	23	10	Low	Low
P13	0	64	5	Low	High
P14	2	70	12	Low	High
P15	9	118	46	High	High
P16	7	53	39	High	Medium

## 4 Discussion

### 4.1 Main findings

From the population interviewed, different considerations may be drawn.

There is widespread recognition of pollution sources, with industries, transportation, heating systems, and animal farming commonly cited. According to the “INventario EMissioni ARia” (INEMAR) (23), the air emissions inventory, report for the Lombardy region in 2021, non-industrial combustion (i.e., mostly wood combustion) contributes 52.3 and 44.7% to PM2.5 and PM10 emissions, respectively, while road transportation contributes 8.4 and 22.5%, respectively. Similar trends are observed for NO_x_ emissions, whereas agriculture accounts for 95.5% of NH_3_ emissions. Sources of CO_2_ and CO emissions are diverse, including energy production, industrial and non-industrial combustion, and road transport. Notably, the participants of the present study mentioned heating systems but not cooling systems, despite air conditioning being equally polluting as heating. This discrepancy may be attributed to the test taking place in autumn, potentially biasing respondents toward heating-related responses based on seasonal habits. Another consideration is that in some cases, we found general terms such as “smoke” to indicate a source of pollution, which is reflected also in literature, as previously explained.

Although a generally strong understanding was evident regarding the health effects of pollution, it was unexpected that not all participants mentioned respiratory issues in connection with air pollution, despite this being an immediate implication. However, several individuals emphasized the impact of pollution on psycho-physical health, aligning with contemporary research on air pollution exposure. As an example, numerous studies are investigating indoor air pollution and the sick building syndrome, characterized by symptoms such as fatigue, headaches, and decreased cognitive functions, which are linked, among other factors, to air pollution (24, 25).

The knowledge about air quality monitoring technology was not so solid in the tested population. In the context of the previously reported project InformAria, a survey was delivered to 788 respondents in Milan in May 2023 (15). A question was “Do you know about the quality of the air you are breathing?” and 82% responded yes, but only 13% declare to monitor them continuously, 24% consult data twice/three times a week, while 46% reported to access information seldom. Also, an investigation about the perceived level of information about air quality reveals that Italy ranks 6th from the bottom (26).

Generally, the population tested in our trial refers to using internet to search for air quality data (60%), while an equal number of people refer to using municipality or ARPA website and smartphone application. This was reflected also in the results found by the InformAria survey.

A notable shift in awareness was observed among the participants of this study following their interaction with the air pollution monitoring device. Despite the relatively small sample size, this suggests that a technological tool such as the one presented can effectively enhance awareness and cultivate a more informed citizenry. This, in turn, may lead to greater public support for policies aimed at improving air quality.

Despite most of the sample being composed of engineers, many of the participants rely on sensory cues to evaluate air pollution and, after the walk, 44% still reported relying on sensory cues as indicators of air quality. This reliance, even after having experienced the wearable device real-time data, may be influenced by the specific locations and types of pollution encountered. For instance, encountering strong odors at a gas station where pollution levels are high, or passing by smokers and smelling the odor before observing increased pollution levels on the map, could create a bias. This observation underscores the limited baseline knowledge about air pollution among individuals who only check air quality when it is known to be poor. Thus, a tool like the one presented in this paper could be instrumental in helping citizens understand the dynamic changes in air pollution they encounter in daily life, allowing them to observe variations in air quality that extend beyond what can be perceived through sensory cues.

The tested population demonstrated significant interest in having a device like the one used in the protocol. However, a sense of being overly monitored was noted, potentially resulting in increased stress and anxiety. This phenomenon has already been documented in the literature. For instance, a 2020 study conducted an initial online survey followed by interviews with seven individuals who had used an air pollution monitoring device (27). The study aimed to understand current air quality monitoring practices and personal preferences regarding such technologies. Participants expressed differing attitudes toward monitoring: some wanted to be aware of air quality in their immediate environment to take necessary actions, while others felt indifferent or anxious about knowing, even finding some comfort in remaining uninformed. The study encapsulated this tension with the phrase “To Monitor or Not to Monitor,” linking it to the idea that having the ability to take tangible actions to reduce air pollution exposure or contribute to lower pollution levels can significantly impact one’s experience of monitoring. A recent four-week study conducted in Germany (28) displayed real-time CO_2_ and TVOC levels in office environments and demonstrated that ventilation strategies based on pollution data were successfully implemented. This suggests that when monitoring provides clear and practical actions that are easy to adopt, such as strategic ventilation to improve indoor air quality, individuals are more likely to follow these recommendations.

In this context, considering that the test population comprises technology enthusiasts who are already familiar with receiving extensive data, providing actionable information could help reduce feelings of being overwhelmed and alleviate concerns about excessive monitoring. Empowering individuals with practical steps to protect themselves from pollution and promote cleaner air could mitigate anxiety and foster a sense of control. The anxiety associated with over-monitoring is linked not only to the uncertainty about how to effectively reduce exposure but also to the concern that, even with appropriate measures in place, individuals may still experience high levels of exposure due to the actions—or inaction—of others. A 2020 study conducted in Chile involved 36 households testing an indoor environmental monitor aimed at reducing exposure to pollution from wood-burning stoves (29). Although participants could view real-time feedback and took actions such as refraining from using stoves to reduce indoor air pollution, these efforts were not always effective. The high levels of indoor pollution persisted due to outdoor pollution infiltrating the indoor environment, leading to a sense of frustration and perceived unfairness, as participants remained highly exposed despite their efforts. This indicates the need for a combined approach to address the issue: a bottom-up strategy that focuses on raising awareness and promoting actions at the individual level, coupled with a top-down approach that involves implementing structural policies and community-based strategies to mitigate pollution on a broader scale.

Regarding behavioral responses relevant to air pollution, a study dating back to 1991 (30) identified them as actions to reduce air pollution (e.g., driving less), and actions to self-protect from air pollution (e.g., avoid some outdoor activity). Interestingly, discussions about behavioral changes related to air pollution focused solely on protective actions rather than actions to actively improve air quality, such as substantial changes in one’s own lifestyle. This may be because self-protection is perceived as a more immediate and achievable action, offering individuals a sense of control over their own well-being. In contrast, efforts to reduce pollution production are often seen as overwhelming and complex, requiring collective action and systemic changes that go beyond the influence of a single person. As a result, people may prioritize self-protective behaviors, such as wearing masks or using air purifiers, because they provide more tangible and direct benefits, whereas the impact of individual efforts to reduce overall pollution may seem less immediate or significant.

Additionally, interviews revealed strategies for crisis management previously described in the Introduction, such as the perception that the local air quality is better than elsewhere or resignation to the situation.

Finally, insights can be derived from the parallel analysis of participants’ responses and their PM2.5 exposure levels. The findings suggest that individuals with the highest median exposure to PM2.5 were more likely to exhibit increased awareness, greater interest in the monitoring device, and a stronger willingness to adopt behavioral changes in response to pollution levels.

### 4.2 Study limitations and future work

One of the main limitations of this study is the small sample size and the homogeneity of the participants. Despite this, the review article by Noël et al. (1) cites several studies with similar sample sizes. The semi-structured interview design used in our research is time-consuming, often resulting in smaller sample sizes, as surveys are typically preferred for larger groups. Nonetheless, we plan to involve a larger population in future studies.

The homogeneity of the sample was intentional. This approach allowed us to assess interest, awareness shifts, and willingness to change behavior without considering potential barriers that some individuals might face when interacting with a technological tool like the one used in this protocol. Interestingly, our findings revealed a pattern: participants exposed to higher levels of pollution tended to provide consistent feedback. This suggests that feedback may be more closely related to the pollution levels experienced rather than the participants’ technological enthusiasm. Therefore, in the next phase of our research, we aim to engage a more diverse population. Another limitation relates to the lack of detailed demographic information about the participants, such as their living environments, origins, and habits. Future studies should aim to collect more comprehensive data on the environments in which individuals live and work to gain deeper insights into their awareness and behaviors.

Another limitation is the short duration of the testing. In future studies, we plan to extend the usage period to several weeks, thereby increasing the likelihood of participants encountering high pollution levels and high variability of pollution. This extended timeframe will provide deeper insights into the issue and help determine whether expressed willingness to change translates into actual behavior change regarding pollution exposure.

Additionally, the app’s current visual design only displays the concentration of the monitored pollutants. Future iterations should incorporate another layer of communication to help users better understand the scale of pollution. Another feature that can facilitate engagement with the topic is the use of personal exposure history. This feature enables individuals to track how changes in behavior, such as taking protective actions or choosing less polluted routes, impact their exposure over time. Moreover, as our findings align with existing literature, providing more actionable information will enhance user engagement.

Lastly, a potential source of bias must be acknowledged, as questions were administered in person by the project operators. However, participants were encouraged to express their thoughts sincerely, as both positive and negative feedback are essential for guiding the next steps of our research.

## 5 Conclusion

This study sheds light on the intricate dynamics of public perception regarding air pollution sources, health implications, and monitoring technologies. Despite the predominantly technology-oriented background of the participants, reliance on sensory cues for air quality assessment was prevalent, underscoring the need for comprehensive education on pollution monitoring methods.

Our findings reveal a robust understanding of pollution sources, with industries, transportation, and agricultural activities prominently recognized. However, a surprising lack of unanimous association between air pollution and respiratory issues among some participants highlights that targeted educational campaigns may be helpful to bridge knowledge gaps.

Moreover, our study highlights the transformative potential of technological interventions in enhancing public awareness of air quality issues. Interaction with air pollution monitoring devices elicited a notable shift in participant awareness, suggesting that such tools can play a pivotal role in empowering citizens to advocate for air quality improvement initiatives.

Despite the enthusiasm for adopting air pollution monitoring technologies, concerns about over-monitoring leading to increased stress and anxiety were raised. Addressing these concerns requires a nuanced approach, emphasizing the provision of actionable information on protective measures against pollution and strategies for promoting better air quality.

## Data Availability

The raw data supporting the conclusions of this article will be made available by the authors, without undue reservation.

## References

[1] Noël C Vanroelen C Gadeyne S. Qualitative research about public health risk perceptions on ambient air pollution. A review study. SSM Popul Health. (2021) 15:100879. doi: 10.1016/J.SSMPH.2021.100879, PMID: 34355057 PMC8325091

[2] Dimitriou A Christidou V. Pupils’ understanding of air pollution. J Biol Educ. (2007) 42:24–9. doi: 10.1080/00219266.2007.9656103

[3] WHO. (2022). WHO publishes new global data on the use of clean and polluting fuels for cooking by fuel type Available at: (https://www.who.int/news/item/20-01-2022-who-publishes-new-global-data-on-the-use-of-clean-and-polluting-fuels-for-cooking-by-fuel-type). PMC9280531 35256505

[4] McCarron A Semple S Swanson V Braban CF Gillespie C Price HD. “I have to stay inside …”: experiences of air pollution for people with asthma. Health Place. (2024) 85:103150. doi: 10.1016/J.HEALTHPLACE.2023.103150, PMID: 38064920

[5] Bickerstaff K Walker G. The place(s) of matter: matter out of place - public understandings of air pollution. Prog Hum Geogr. (2003) 27:45–67. doi: 10.1191/0309132503PH412OA

[6] Day R. Place and the experience of air quality. Health Place. (2007) 13:249–60. doi: 10.1016/J.HEALTHPLACE.2006.01.002 16500135

[7] Xu J Chi CSF Zhu K. Concern or apathy: the attitude of the public toward urban air pollution. J Risk Res. (2017) 20:482–98. doi: 10.1080/13669877.2015.1071869

[8] Nicolau JL Mellinas JP Martin-Fuentes E. The halo effect In: Buhalis D, editor. Encyclopedia of tourism management and marketing. Cheltenham: Edward Elgar Publishing (2022)

[9] Hill RJ Fishbein M Ajzen I. Belief, attitude, intention, and behavior: an introduction to theory and research. Contemp Sociol. (1977) 6:244. doi: 10.2307/2065853

[10] Hatfield J Job RFS. Optimism BIAS about environmental degradation: the role of the range of impact of precautions. J Environ Psychol. (2001) 21:17–30. doi: 10.1006/JEVP.2000.0190

[11] Ramírez AS Ramondt S Van Bogart K Perez-Zuniga R. Public awareness of air pollution and health threats: challenges and opportunities for communication strategies to improve environmental health literacy. J Health Commun. (2019) 24:75–83. doi: 10.1080/10810730.2019.1574320, PMID: 30730281 PMC6688599

[12] Bernasconi S Angelucci A Aliverti A. A scoping review on wearable devices for environmental monitoring and their application for health and wellness. Sensors. (2022) 22:5994. doi: 10.3390/S22165994 36015755 PMC9415849

[13] Kureshi RR Thakker D Mishra BK Barnes J. From raising awareness to a Behavioural change: a case study of indoor air quality improvement using IoT and COM-B model. Sensors. (2023) 23:3613. doi: 10.3390/S23073613 37050669 PMC10098860

[14] Rickenbacker H Brown F Bilec M. Creating environmental consciousness in underserved communities: implementation and outcomes of community-based environmental justice and air pollution research. Sustain Cities Soc. (2019) 47:101473. doi: 10.1016/J.SCS.2019.101473

[15] Cambia Aria-Comune di Milano. (2022). InformARIA-Milano. Available at: (https://www.comune.milano.it/web/milano-cambia-aria/progetti/informaria).

[16] Haddad H de Nazelle A. The role of personal air pollution sensors and smartphone technology in changing travel behaviour. J Transp Health. (2018) 11:230–43. doi: 10.1016/J.JTH.2018.08.001

[17] Bernasconi S Angelucci A Rossi A Aliverti A. A wearable system for personal air pollution exposure: a walk-about in Milan. Eur Respir J. (2023) 62:PA2908. doi: 10.1183/13993003.CONGRESS-2023.PA2908

[18] Bernasconi S. Angelucci A. Aliverti A., (2024). Evaluation of a new wearable device for indoor and outdoor environmental monitoring. In 2024 IEEE international workshop on sport, technology and research (STAR). Lecco, Italy: IEEE. 252–257.

[19] Braun V Clarke V. Using thematic analysis in psychology. Qual Res Psychol. (2006) 3:77–101. doi: 10.1191/1478088706QP063OA

[20] Consulta il meteo su iPhone. (2024). Supporto Apple (IT). Available at: (https://support.apple.com/it-it/guide/iphone/iph1ac0b35f/ios).

[21] ARPA Lombardia. (2024). Home Page - ARPA Lombardia. Available at: (https://www.arpalombardia.it/).

[22] Meteo Italia. (2024). Previsioni del tempo per tutti i comuni - Weather Italy. Available at: (https://www.ilmeteo.it/Italia).

[23] Principali risultati Inventario (2021). Qualitative research about public health risk perceptions on ambient air pollution. A review study. Available at: (https://www.inemar.eu/xwiki/bin/view/InemarDatiWeb/Principali+risultati+2021). 10.1016/j.ssmph.2021.100879 PMC8325091 34355057

[24] Hou J Sun Y Dai X Liu J Shen X Tan H. Associations of indoor carbon dioxide concentrations, air temperature, and humidity with perceived air quality and sick building syndrome symptoms in Chinese homes. Indoor Air. (2021) 31:1018–28. doi: 10.1111/INA.12810, PMID: 33620091

[25] Mentese S Mirici NA Elbir T Palaz E Mumcuoğlu DT Cotuker O. A long-term multi-parametric monitoring study: indoor air quality (IAQ) and the sources of the pollutants, prevalence of sick building syndrome (SBS) symptoms, and respiratory health indicators. Atmos Pollut Res. (2020) 11:2270–81. doi: 10.1016/J.APR.2020.07.016

[26] Attitudes of Europeans towards Air Quality. (2022). Eurobarometer survey. Available at: (https://europa.eu/eurobarometer/surveys/detail/2660).

[27] Sakhnini N. Yu J.E. Jones R.M. Chattopadhyay D., (2020). Personal air pollution monitoring technologies: user practices and preferences. In HCI International 2020 - Late breaking papers: user experience design and case studies, HCI International Conference: Copenhagen, Denmark

[28] Bitomsky L. Meindl O. Schmidt M. Regal C., (2020). The effect of real-time feedback on indoor environmental quality. In 15th International Conference on Wirtschaftsinformatik. Potsdam: Wirtschaftsinformatik

[29] Boso À Álvarez B Oltra C Garrido J Muñoz C Hofflinger Á. Out of sight, out of mind: participatory sensing for monitoring indoor air quality. Environ Monit Assess. (2020) 192:104. doi: 10.1007/S10661-019-8058-Z, PMID: 31915931

[30] Skov T Cordtz T Jensen LK Saugman P Schmidt K Theilade P. Modifications of health behaviour in response to air pollution notifications in Copenhagen. Soc Sci Med. (1991) 33:621–6. doi: 10.1016/0277-9536(91)90220-7, PMID: 1720575

